# PCR & Go: A Pre-installed Expression Chassis for Facile Integration of Multi-Gene Biosynthetic Pathways

**DOI:** 10.3389/fbioe.2020.613771

**Published:** 2021-01-14

**Authors:** Mingming Qi, Bei Zhang, Lihong Jiang, Saijuan Xu, Chang Dong, Yi-Ling Du, Zhan Zhou, Lei Huang, Zhinan Xu, Jiazhang Lian

**Affiliations:** ^1^Key Laboratory of Biomass Chemical Engineering of Ministry of Education, College of Chemical and Biological Engineering, Zhejiang University, Hangzhou, China; ^2^Center for Synthetic Biology, College of Chemical and Biological Engineering, Zhejiang University, Hangzhou, China; ^3^School of Bioengineering, Dalian University of Technology, Dalian, China; ^4^Institute of Pharmaceutical Biotechnology, Zhejiang University School of Medicine, Hangzhou, China; ^5^Institute of Drug Metabolism and Pharmaceutical Analysis, College of Pharmaceutical Sciences, Zhejiang University, Hangzhou, China

**Keywords:** multiplex genome integration, CRISPR/Cas9, metabolic engineering, carotenoid biosynthesis, *Saccharomyces cerevisiae*

## Abstract

The introduction of multi-gene metabolic pathways is generally the first step for the construction of microbial cell factories and plays an essential role in metabolic engineering and synthetic biology. Here, we developed a “PCR & Go” system for facile integration and assembly of multi-gene pathways into the chromosome of *Saccharomyces cerevisiae*. The core component of the “PCR & Go” system was an expression chassis, where eight promoter/terminator pairs were pre-installed into the yeast chromosome and PCR amplified gene fragments could be inserted directly for functional expression. In combination with the CRISPR/Cas9 system and a gRNA plasmid library, the β-carotene (three genes), zeaxanthin (four genes), and astaxanthin (five genes) biosynthetic pathways were integrated and assembled into the yeast genome with an efficiency of ~93, ~85, and 69%, respectively, using PCR amplified gene fragments with ~40 bp homology arms in a single step. Therefore, the “PCR & Go” system can be used for fast construction of yeast cell factories harboring multi-gene pathways with high efficiency and flexibility.

**Graphical Abstract:**
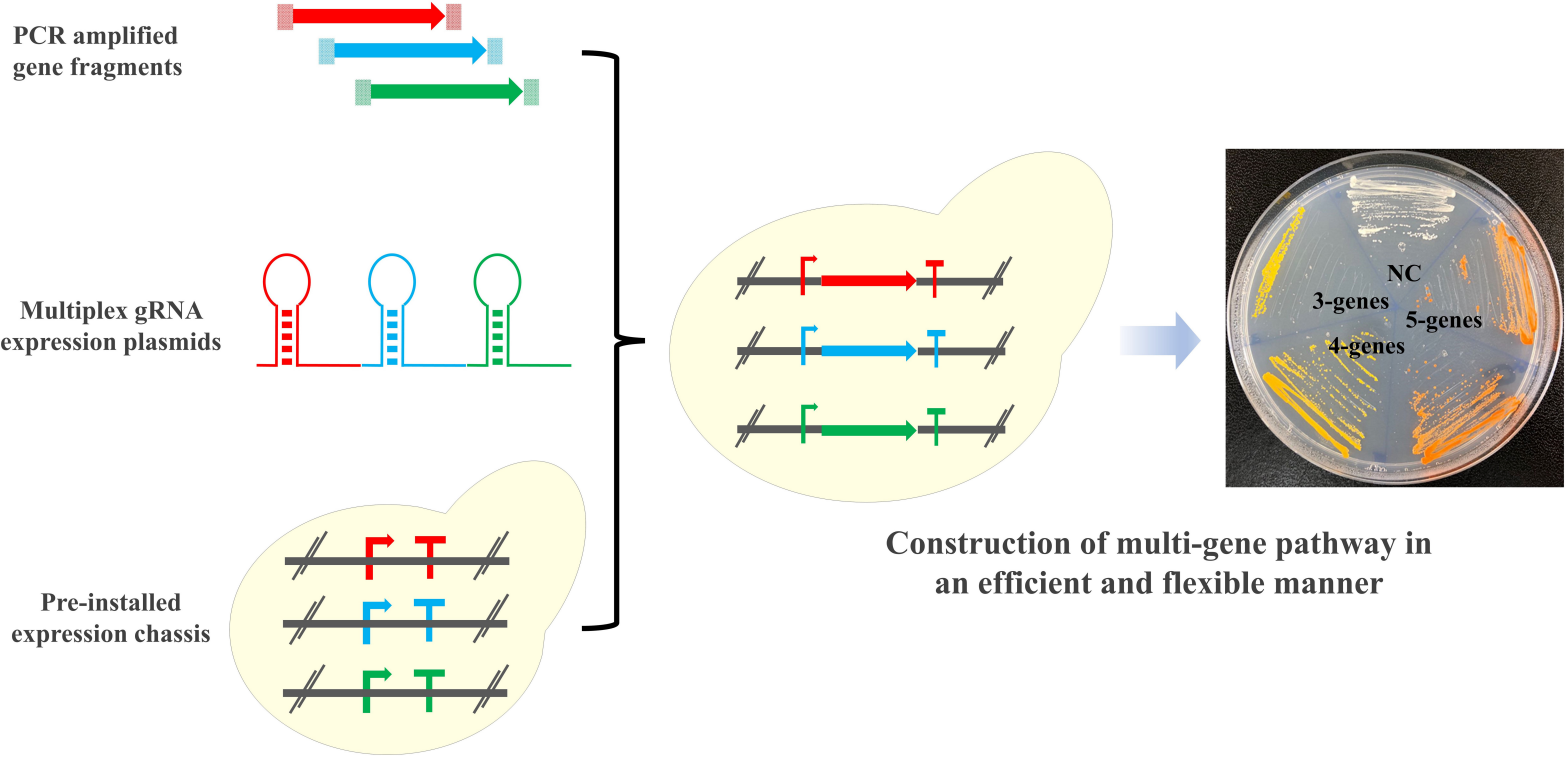
Schematic overview of the “PCR & Go” system. PCR amplified gene fragments and multiplex gRNA expression plasmids were co-transformed to the pre-installed expression chassis for facile integration and assembly of multi-gene pathways into the chromosome of *Saccharomyces cerevisiae*.

## Introduction

There have been growing interests in using microbial cell factories to synthesize fuels, chemicals, and pharmaceutics in recent years (Nielsen and Keasling, 2016; Lian et al., 2018c; Yang et al., 2019). The synthesis of non-native compounds generally involves the reconstruction of heterologous multi-gene metabolic pathways. Episomal plasmids and chromosomal integration are commonly employed for heterologous expression of genes and pathways in *Saccharomyces cerevisiae*. While the plasmid system suffers from low genetic stability and high maintenance cost, the integration of pathway genes into the genome is preferred for biotechnological applications (Tyo et al., 2009; Da Silva and Srikrishnan, 2012).

**Figure 1:**
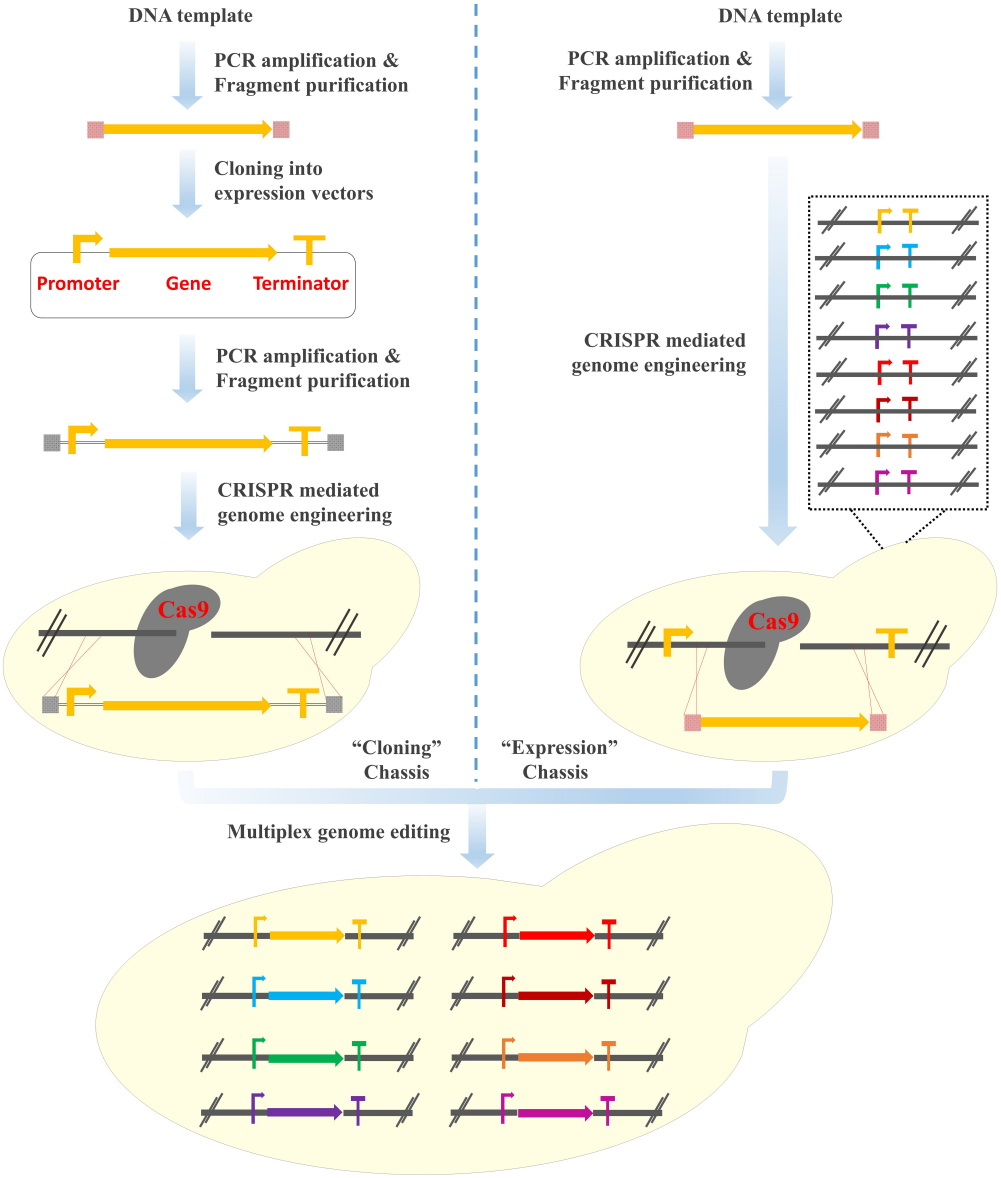
Design of the “PCR & Go” system for the integration of multi-gene pathways in *S. cerevisiae*. (Left panel) Multiplex genome integration using the CRISPR/Cas9 system. Tedious process of plasmid construction is generally required. (Right panel) Multiplex genome integration using the “PCR & Go” system. PCR amplified gene fragments are integrated into the expression chassis directly.

The Clustered Regularly Interspaced Short Palindromic Repeats (CRISPR)/CRISPR-associated systems (Cas) has revolutionized the genome editing field (Hsu et al., 2014; Shalem et al., 2015; Zhang et al., 2020) and become the thumb of rule for multiplex genome engineering of yeast cell factories (DiCarlo et al., 2013; Lian et al., 2018b). For example, Ronda et al. developed the CrEdit (CRISPR/Cas9 mediated genome editing) system for simultaneous integration of the three pathway genes involved in β-carotene biosynthesis (84% integration efficiency with 500 bp homology arms) (Ronda et al., 2015). Jessop-Fabre et al. developed the EasyClone-Marker-Free vector toolkit for multiplex genome integration, with a targeting efficiency of 90–100% and 60–70% for single and triple loci editing, respectively (Jessop-Fabre et al., 2016). Similarly, Shi et al. designed Di-CRISPR (Delta Integration CRISPR/Cas9) to achieve multi-copy and marker-less integration of DNA constructs ranging from 8 kb to 24 kb with high efficiencies in a single step (Shi et al., 2016). Recently, Bourgeois et al. constructed a Landing Pad system, where synthetic DNA fragments functioning as the homologous recombination arms with different copy numbers were pre-inserted into the chromosomes and the heterologous gene expression cassettes (promoter-gene-terminator) could be integrated into the yeast genome with precisely controlled copy numbers (from one to four copies) (Bourgeois et al., 2018). However, tedious process of plasmid construction (i.e., the cloning of the target genes into plasmids together with the transcription and translation elements) is generally required (Figure 1, Left panel). In addition, repetitive steps of PCR amplification of the regulatory elements and gene coding sequences not only lead to a waste of resources, but also dramatically increase the chance of introducing undesirable mutations.

From the perspective of heterologous gene expression on episomal plasmids, expression vectors have been widely used, such as the pET and pESC series vectors for *Escherichia coli* and *S. cerevisiae*, respectively (Hausjell et al., 2018). Different with the cloning vectors, expression vectors were constructed by pre-assembling transcription and translation elements (i.e., promoters, terminators, and ribosome binding sites), where heterologous genes can be inserted directly to achieve functional expression. From this point of view, all the currently used synthetic biology hosts should be considered as “cloning” chassis. Expression chassis can be established by inserting promoters and terminators into the well-characterized regions of the chromosome. Thanks to the high homologous recombination efficiency in *S. cerevisiae*, efficient expression of the heterologous genes can be achieved by integrating the coding sequences with 40 bp homology arms, which can be generated by PCR directly. In other words, no plasmid construction and repetitive PCR amplification is required and the time for the construction of heterologous metabolic pathways can be significantly shortened. In addition, synthetic linkers with Cas9 targeting sequences (equivalent to multiple cloning sites of the expression vectors) are inserted between promoters and terminators, enabling the integration of heterologous genes in a CRISPR-assisted multiplex manner. These pre-installed gene expression elements function as the “gates” of the heterologous genes to enter the genome and the combination with the CRISPR/Cas9 system enables the simultaneous opening of multiple “gates” in an efficient and precise manner (Figure 1, Right panel). In this case, multi-gene biosynthetic pathways can be integrated and assembled into the genome by using PCR amplified fragments directly.

In the present study, we aimed to develop a “PCR & Go” system based on expression chassis for direct integration and assembly of multi-gene biosynthetic pathways into the yeast genome by CRISPR/Cas9. First, we chose and inserted eight promoter/terminator pairs together with CRISPR targeted synthetic linkers into the previously characterized genomic loci. Then, we integrated the *mCherry* coding sequences amplified by PCR into these pre-installed promoter-linker-terminator regions to test the efficiency of the “PCR & Go” system. Finally, we evaluated the efficiency as well as flexibility of the “PCR & Go” system by constructing yeast strains that were able to produce β-carotene (three genes), zeaxanthin (four genes), and astaxanthin (five genes) using PCR amplified gene fragments with ~40 bp homology arms in a single step.

## Results

### Construction of an Expression Chassis of *S. cerevisiae*

In order to establish the “PCR & Go” system for fast and efficient integration of heterologous metabolic pathways, we firstly chose eight chromosomal integration loci that had been previously characterized to enable high level expression of heterologous genes (Supplementary Table 1) (Reider Apel et al., 2017). Using *TDH3*p*-mCherry-CYC1*t as the reporter, we achieved nearly 100% integration efficiency and the expression levels were comparably high (Supplementary Figure 1), indicating all the eight loci could be used for subsequent studies. In addition, we selected eight strong constitutive promoters (*GPM1*p, *TDH3*p, *ENO2*p, *HSP104*p, *TEF1*p, *SSA1*p, *CCW12*p, and *HHF2*p) and terminators (*ADH1*t, *CYC1*t, *PGK1*t, *CPS1*t, *TEF1*t, *PRM9*t, *PRM5*t, and *IDP1*t) commonly used in *S. cerevisiae* (Sun et al., 2012; Curran et al., 2013; Lian and Zhao, 2015) (Supplementary Table 2). Orthogonal synthetic linkers (Lian et al., 2019) containing unique gRNA target sequences were inserted in between of each promoter and terminator (Supplementary Table 3). The expression chassis (MQ009, Supplementary Table 4) was established by integrating the eight synthetic DNA fragments containing promoter-linker-terminator into the above-mentioned genomic loci.

### Establishment of a “PCR & Go” Toolkit

To make the “PCR & Go” system a more powerful tool for facile integration of multi-gene biosynthetic pathways, we then created a gRNA plasmid library (Table 1), including those with different gRNA targeting sequences, varied number of gRNA expression cassettes, and several selection markers to maintain the plasmids. Our customized engineering goals could be satisfied by choosing different combinations of the gRNA plasmids. For example, we could integrate and assemble a five-gene biosynthetic pathway by co-transforming p423-sgRNA-M1-M2-M3 and p426-sgRNA-M4-M5 together with the donor DNA fragments in a single step. In other words, multiplex genome integration could be achieved in an efficient and precise manner.

**Table 1:** List of gRNA plasmids in the “PCR & Go” toolkit.

	**Vector backbones**	
	**pRS423 (*HIS3*)**	**pRS426 (*URA3*)**
Mono-gRNA plasmids	p423-sgRNA-M1 p423-sgRNA-M2 p423-sgRNA-M3 p423-sgRNA-M4 p423-sgRNA-M5 p423-sgRNA-M6 p423-sgRNA-M7 p423-sgRNA-M8	p426-sgRNA-M4 p426-sgRNA-M6 p426-sgRNA-M8
Dual-gRNA plasmids	p423-sgRNA-M1-M2 p423-sgRNA-M2-M5 p423-sgRNA-M4-M5 p423-sgRNA-M4-M8 p423-sgRNA-M5-M8 p423-sgRNA-M7-M8	p426-sgRNA-M4-M5 p426-sgRNA-M4-M6 p423-sgRNA-M4-M8 p426-sgRNA-M5-M8
Triple-gRNA plasmids	p423-sgRNA-M1-M2-M3 p423-sgRNA-M1-M2-M4	p426-sgRNA-M2-M3-M5 p426-sgRNA-M4-M5-M6 p426-sgRNA-M3-M5-M8
Quadruple-gRNA plasmids	p423-sgRNA-M1-M2-M3-M4	Not constructed

### Evaluation of the “PCR & Go” System Using a Reporter Gene

**Figure 2:**
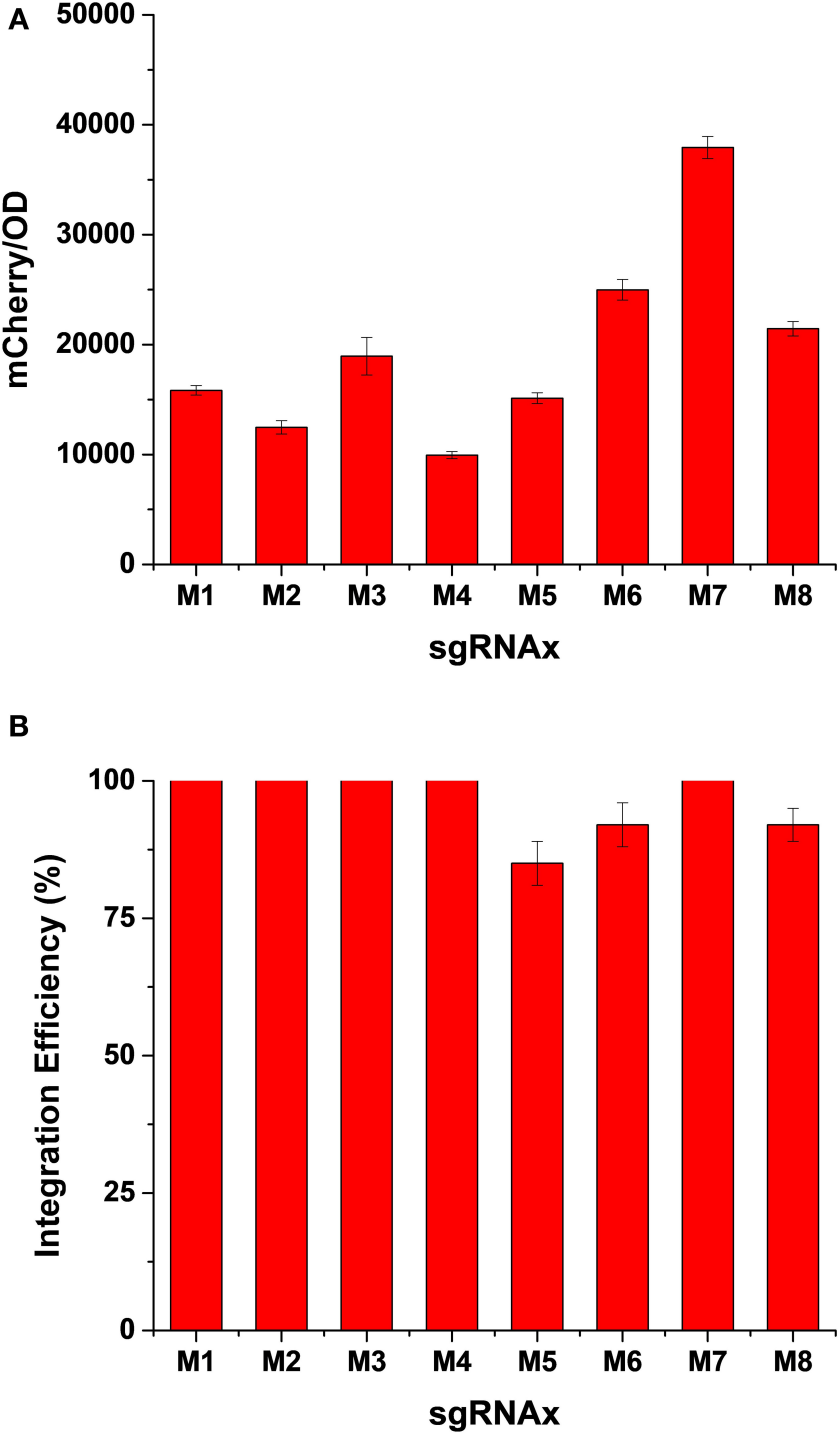
Evaluation of expression level **(A)** and integration efficiency **(B)** of heterologous genes in different genomic loci of the expression chassis. The integration efficiency was calculated as number of colonies with *mCherry* fluorescence/total number of colonies tested. Error bars represented the mean ± s.d. of biological triplicates.

As a proof-of-concept of the “PCR & Go” system, we integrated the *mCherry* coding sequences amplified by PCR with 40 bp homology arms into the pre-installed synthetic DNA fragments. After co-transformation of the gRNA plasmid and the PCR amplified fragment into the expression chassis, we randomly picked eight colonies from each plate to evaluate both the expression level and integration efficiency of heterologous genes. As shown in Figure 2A, while *mCherry* fluorescence intensities were comparable in most cases, the integration into M6 (*SSA1*p-*PRM9*t) and M7 (*CCW12*p-*PRM5*t) loci resulted in much higher expression levels, probably due to a combined effect of genomic loci and promoter strength on gene expression. Nevertheless, we achieved decent integration efficiency for all constructs, in particular 100% integration efficiency when using gRNA-M1, gRNA-M2, gRNA-M3, gRNA-M4, and gRNA-M7 (Figure 2B).

### “PCR & Go” for Facile Construction of Yeast Cell Factories With Multi-Gene Biosynthetic Pathways

**Figure 3:**
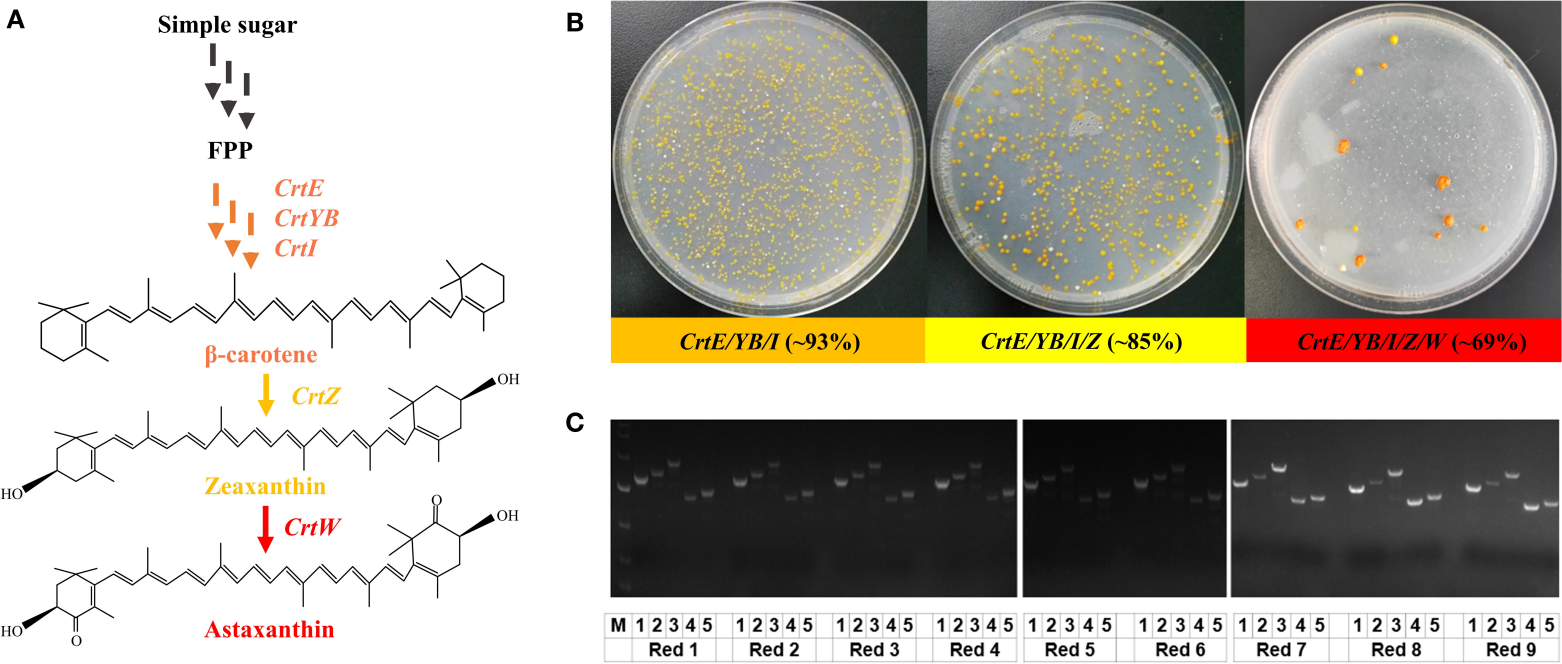
“PCR & Go” system for the construction of yeast cell factories for β-carotene, zeaxanthin, and astaxanthin. **(A)** Overview of the carotenoid biosynthetic pathway. CrtE: geranylgeranyl diphosphate synthase; CrtYB: phytoene synthase/lycopene cyclase; CrtI: phytoene desaturase; CrtZ: β-carotene hydroxylase; CrtW: β-carotene ketolase. Multiple arrows indicate multiple reaction steps. **(B)** Integration efficiency of the three-gene, four-gene, and five-gene pathways using the “PCR & Go” system. 1.5 μg of the PCR amplified gene fragments with 40 bp homology arms together with the corresponding gRNA plasmid(s) were co-transformed into the expression chassis and the integration efficiency was calculated based on both genotypic (diagnostic PCR) and phenotypic (pigment formation) results. The integration efficiency of the β-carotene pathway was calculated as the percentage of pigmented colonies (number of orange colonies/total number of colonies on the agar plate). The integration efficiency of the astaxanthin pathway was calculated as the number of colonies with correct PCR patterns for five genes/total number of colonies on the agar plate. **(C)** Genotyping of the nine colonies in red on the astaxanthin agar plate. Diagnostic PCR results for the yellow colonies and white colony were provided in Supplementary Figure 2. 1, 2, 3, 4, 5 denoted *CrtE, CrtI, CrtYB, CrtW*, and *CrtZ* integrated into the expression chassis, respectively.

Finally, with the establishment of the “PCR & Go” system, we attempted to evaluate its efficiency and flexibility in the construction of yeast cell factories. We chose the carotenoid biosynthetic pathways (Shao et al., 2009) for demonstration (Figure 3A), with β-carotene biosynthesis as an example for the integration and assembly of three-gene pathways (*CrtE, CrtYB*, and *CrtI*), zeaxanthin for four-gene pathways (*CrtE, CrtYB, CrtI*, and *CrtZ*), and astaxanthin for five-gene pathways (*CrtE, CrtYB, CrtI, CrtZ*, and *CrtW*). By co-transforming p423-sgRNA-M1-M2-M3 and PCR amplified *CrtE, CrtYB*, and *CrtI* fragments with 40 bp homology arms, the β-carotene-producing yeast strain was constructed in a single step with an efficiency higher than 93% (Figure 3B). Noteworthy, the use of different combinations of gRNAs (i.e. M2-M3-M5 and M3-M5-M8) resulted in slightly lower integration efficiency of the same three-gene biosynthetic pathway (Supplementary Figure 3). Therefore, M1, M2, and M3 were chosen for the integration of *CrtE, CrtYB*, and *CrtI*, respectively, in the following studies. The integration of an additional DNA fragment (*CrtZ*) by p423-sgRNA-M1-M2-M3-M4 resulted in the construction of zeaxanthin-producing yeast strain with an efficiency approaching 85%. Similarly, the astaxanthin biosynthetic pathway was integrated and assembled in a single step using the “PCR & Go” system. After co-transformation of p423-sgRNA-M1-M2-M3, p426-sgRNA-M4-M5, as well as PCR amplified *CrtE, CrtYB, CrtZ, CrtW*, and *CrtI* fragments, we obtained 13 colonies on the agar plate in total (Figure 3B): nine colonies in red (potential astaxanthin), three colonies in yellow (potential β-carotene or zeaxanthin), and only one colony in white (no carotenoid production). We then genotyped all the 13 clones via diagnostic PCR and found that all five genes were correctly integrated into the desirable genomic loci in the red colonies (Figure 3C), the size of *CrtW* was larger than expected in all the three yellow colonies, while a truncated version of *CrtE* was integrated in the white colony (Supplementary Figure 2). The genotype of the yeast strains was consistent with the observed phenotypes: five-gene pathway integration resulted in the formation of the red pigment astaxanthin, the integration of *CrtE, CrtYB, CrtI*, and *CrtZ* resulted in the biosynthesis of zeaxanthin (yellow pigment), and the wrong integration pattern of *CrtE* (encoding the first enzyme in carotenoid biosynthesis) resulted in no pigment formation. The biosynthesis of β-carotene and astaxanthin were verified by HPLC analysis (Supplementary Figure 4). Noteworthy, although *CrtW* and *CrtE* were not correctly integrated in the yellow colonies and the white colony, respectively, we obtained PCR amplicons for all the clones (Figure 3C, Supplementary Figure 2). Diagnostic PCR results indicated that all these five genomic loci were successfully edited with 100% efficiency and at least four genes were correctly integrated by the CRISPR/Cas9 system. The integration efficiency of the “PCR & Go” system might be further enhanced by improving the quality of the PCR amplified gene fragments. Overall, the five genes involved in astaxanthin biosynthesis were simultaneously integrated to the expression chassis with an efficiency of ~69% (9 out of 13 colonies). In addition, no colonies lost the ability to synthesize astaxanthin after serial transfer in non-selective medium for more than 40 generations, indicating the stability of the genome-integrated strains.

## Discussion

Recently, an increasing number of high-value non-native compounds have been synthesized with microbial cell factories, highlighting the significance of genetic manipulation particularly multiplex genome editing technologies. Owing to the high efficiency and ease of use, CRISPR/Cas has been generally regarded as the must-have technology for the metabolic engineering and microbial biotechnology communities (Lian et al., 2018b; Zhang et al., 2020). Therefore, a panel of CRISPR-aided methods have been established to integrate multi-gene biosynthetic pathways in a multiplex and/or multi-copy manner, such as CrEdit (Ronda et al., 2015), CasEMBLR (Jakočiunas et al., 2015), the EasyClone-Marker-Free vector toolkit (Jessop-Fabre et al., 2016), Di-CRISPR (Shi et al., 2016), and Landing Pad (Bourgeois et al., 2018). In contrast to these previously established systems, the “PCR & Go” system developed in the present study achieved efficient integration of five heterologous genes simultaneously with only 40 bp homologous arms. The “PCR & Go” system further accelerates the strain construction process by skipping most of the molecular cloning steps (i.e., restriction digestion, ligation or DNA assembly, and plasmid amplification and extraction).

Nevertheless, the “PCR & Go” system is far from perfect and should be further developed, be more versatile for the integration and assembly of multi-gene biosynthetic pathways. Although we were able to integrate six genes in a single transformation with decent efficiencies (~50%), we failed to obtain any transformants in several batches, indicating that the transformation efficiency and/or homologous recombination efficiency should be further optimized for integrating more than five genes simultaneously. In addition, only strong and constitutive promoters were considered in the present study, while the expression level of the target genes should be fine-tuned or turned on under specific conditions for metabolic engineering applications (Lian et al., 2018c). Therefore, promoters with different strengths as well as inducible promoters will be additionally installed into the expression chassis in the near future. Leveraging the idea from the Landing Pad system (Bourgeois et al., 2018), promoter-linker-terminator pairs can be pre-installed with different copies and the copy number of the heterologous genes can be precisely manipulated.

In conclusion, we established a “PCR & Go” system for facile integration of multi-gene biosynthetic pathways in *S. cerevisiae*. Due to the pre-installation of transcription and translation elements in the expression chassis, multi-gene pathways can be integrated and assembled into the chromosome using PCR amplified fragments directly, skipping the tedious process of plasmid construction, and minimizing undesirable mutations resulted from repetitive steps of PCR amplification. In combination with the CRISPR/Cas9 system, we were able to construct three-gene, four-gene, and five-gene pathways with an efficiency of ~93, ~85, and ~69%, respectively, using PCR amplified fragments in a single step. Overall, the “PCR & Go” system enables us to integrate multi-gene biosynthetic pathways in an efficient, fast, and flexible manner. In addition, the “PCR & Go” system can be easily expanded to other synthetic biology chassis to facilitate the construction of microbial cell factories.

## Materials and Methods

### Strains and Media

*E. coli* strain DH5α (Transgen Biotech, Beijing, China) was used for gene cloning and plasmid amplification. *E. coli* recombinant strains were cultured in Luria-Bertani broth medium (OXIOD Biotech, London, UK) with 100 μg/mL ampicillin at 37°C. Yeast strains were routinely cultivated in YPD medium containing 10 g/L yeast extract, 20 g/L peptone, and 20 g/L glucose. Recombinant yeast strains were maintained in synthetic complete medium (SED) consisting of 1.7 g/L yeast nitrogen base without amino acids (BD Diagnostics), 1 g/L mono-sodium glutamate, and 0.6 g/L CSM appropriate amino acid drop out mix (MP Biomedicals, Solon, OH, USA). 200 μg/mL G418 (Sangon Biotech, Shanghai, China) was supplemented when necessary. All chemicals were obtained from Sigma-Aldrich (St. Louis, MO, USA) unless specifically mentioned. Restriction enzymes and Q5 DNA polymerase were purchased from New England Biolabs (Ipswich, MA, USA).

### DNA Manipulation and Strain Construction

DNA fragments were amplified via PCR and purified with a Gene JET PCR Purification Kit (Thermofisher Scientific). Plasmids were extracted from *E. coli* using the AxyPrep Plasmid Miniprep Kit (Axygen) according to the manufacturer's instructions. The gRNA sequences were designed using the Benchling CRISPR tool (https://benchling.com/crispr/) and cloned into p423-SpSgH (Lian et al., 2017) and p426-SpSgH (Lian et al., 2019), respectively. All the genetic parts including eight promoters and eight terminators (Supplementary Table 2) were cloned from the genomic DNA of *S. cerevisiae* BY4741. To construct multiple gRNAs expression plasmids, the individual gRNA expression cassettes were pieced together using the Golden-Gate Assembly, as previously described (Lian et al., 2019). The iCas9 expression cassette was amplified from p42H-iCas9 (Lian et al., 2018a) and cloned into the *Sal*I/*Sac*II sites of pRS-KanMX-PmeI-Delta (Du et al., 2012). The resultant plasmid (p41K-INT-iCas9) was linearized by *Pme*I for integration into the chromosome of BY4741 (BY4741-iCas9). All the plasmids and strains used in this study were listed in Supplementary Tables 3, 4, respectively. The corresponding primers were listed in Supplementary Table 5. Oligos used to construct gRNAs were listed in Supplementary Table 6.

### Multiplex Integration of Carotenogenesis Pathway Genes

*CrtI, CrtYB*, and *CrtE* were amplified from the genomic DNA of the yeast strain CEN-Crt (Lian et al., 2017), *CrtZ* was amplified from pRS426-Zea (Lian et al., 2016), and *CrtW* were chemically synthesized for optimal expression in *S. cerevisiae* (Generay Biotech, Shanghai, China). Yeast strains were transformed using the LiAc/SS carrier DNA/PEG method (Gietz and Schiestl, 2007) with some modifications: 1.5 μg of the gRNA plasmids and 1.5 μg of the corresponding donors were co-transformed into the expression chassis (MQ009) followed by heat shock for 60 min at 42°C, and the transformants were selected on the appropriate agar plates. All the DNA sequences of the carotenoid pathway genes were listed in Supplementary Table 7.

### Fluorescence Intensity Measurement

The *mCherry* expressing strains were cultivated in SCD for 2 days and then diluted 5-fold in ddH_2_O for fluorescence assay. The *mCherry* signals were measured using the Tecan Infinite 200 Pro Multimode Reader (Tecan, Switzerland) with an excitation wavelength at 575 nm and an emission wavelength at 610 nm. The fluorescence intensity was normalized to cell density that was determined by measuring the absorbance at 600 nm with the same microplate reader.

## Data Availability Statement

The datasets presented in this study can be found in online repositories. The names of the repository/repositories and accession number(s) can be found in the article/Supplementary Material.

## Author Contributions

MQ and BZ contributed equally to this work. JL and MQ designed the experiments and wrote the manuscript. MQ, BZ, LJ, and CD performed the experiments. All authors read and approved the final manuscript.

## Conflict of Interest

The authors declare that the research was conducted in the absence of any commercial or financial relationships that could be construed as a potential conflict of interest.
